# Chronic cerebral hypoperfusion enhances Tau hyperphosphorylation and reduces autophagy in Alzheimer’s disease mice

**DOI:** 10.1038/srep23964

**Published:** 2016-04-06

**Authors:** Lifeng Qiu, Gandi Ng, Eng King Tan, Ping Liao, Nagaendran Kandiah, Li Zeng

**Affiliations:** 1 Neural Stem Cell Research Lab, Research Department, National Neuroscience Institute, 11 Jalan Tan Tock Seng, Singapore 308433; 2 Calcium Signaling Laboratory, Research Department, National Neuroscience Institute, 11 Jalan Tan Tock Seng, Singapore 308433; 3 Department of Neurology, National Neuroscience Institute, SGH Campus, Singapore 169856; 4 Research Department, National Neuroscience Institute, 11 Jalan Tan Tock Seng, Singapore 308433; 5 Neuroscience & Behavioral Disorders Program, DUKE-NUS Graduate Medical School, Singapore 169857; 6 Neurology Department, National Neuroscience Institute, 11 Jalan Tan Tock Seng, Singapore 308433.

## Abstract

Cerebral hypoperfusion and impaired autophagy are two etiological factors that have been identified as being associated with the development of Alzheimer’s disease (AD). Nevertheless, the exact relationships among these pathological processes remain unknown. To elucidate the impact of cerebral hypoperfusion in AD, we created a unilateral common carotid artery occlusion (UCCAO) model by occluding the left common carotid artery in both young and old 3xTg-AD mice. Two months after occlusion, we found that ligation increases phospho-Tau (p-Tau) at Serine 199/202 in the hippocampus of 3-month-old AD mice, compared to sham-operated AD mice; whereas, there is no change in the wild type (WT) mice after ligation. Moreover, cerebral hypoperfusion led to significant increase of p-Tau in both the hippocampus and cortex of 16-month-old AD mice and WT mice. Notably, we did not detect any change in Aβ_42_ level in either young or old AD and WT mice after ligation. Interestingly, we observed a downregulation of LC3-II in the cortex of aged AD mice and WT mice after ligation. Our results suggest that elevated p-Tau and reduced autophagy are major cellular changes that are associated with hypoperfusion in AD. Therefore, targeting p-Tau and autophagy pathways may ameliorate hypoperfusion-induced brain damage in AD.

Alzheimer’s disease (AD) is the most common cause of dementia worldwide, with the global prevalence estimated at 3.9% in people older than 60 years^1^,^2^; however, AD pathology often coexists with other neurodegenerative and vascular pathologies^3^,^4^. Clinical studies have reported the burden of cerebrovascular disease to be higher in AD than in elderly controls^5^. Autopsy series of patients with AD suggest that the prevalence of vascular pathology ranges from 8% to 35%^6^,^7^. Although the contribution of cerebral large vessel disease to dementia by means of multi-infarct vascular dementia has been widely studied, studies that investigate the contribution of the more prevalent consequences of chronic hypoperfusion, which are manifesting as lacunes and white matter disease, to the pathogenesis of AD remains inadequate^8^,^9^.

With the rising prevalence of AD and failure of anti-amyloid compounds in achieving primary endpoints in AD clinical trials, there is an urgent need to explore other biological factors that can delay AD onset and AD progression^10^. Although cerebrovascular disease (CVD) has been increasingly found to be associated with AD, the underlying mechanism by which CVD contributes to dementia remains unclear^11^. Longitudinal studies that employ amyloid PET scans have demonstrated that the impact of CVD and amyloid to cognitive impairment in AD is independent and additive^12^. This suggests that CVD may be working via non-amyloid pathways to promote the pathogenesis of dementia.

Presently, there are several hypotheses for the underlying mechanisms of CVD in neurodegeneration. These include induction of oxidative stress, Aβ accumulation and aggravation, Tau hyperphosphorylation, synaptic dysfunction, neuronal loss, white matter lesion, and neuroinflammation^13^. Autophagy is a lysosomal degradative process to recycle cellular waste and eliminate potentially toxic damaged organelles and protein aggregates. The involvement of autophagy in ischemic brain^14^ and the defective autophagy in neurodegenerative diseases like AD have been described recently^15^. These defects are considered primary factors contributing to disease pathogenesis rather than being mainly secondary pathological consequences of cellular dysfunction^16^,^17^. Nevertheless, none of the studies reported the role of autophagy in AD + CVD. Since there are more than 60% of the AD patients suffering from cerebral hypoperfusion^18^, this dual pathology implicates that autophagy deficiency is highly likely to be a primary cause of the progression of AD. In addition, in spontaneously hypertensive rat models, an increase in amyloid beta (Aβ) was observed in 20- to 24-week-old animals, and hyperphosphorylated Tau (p-Tau) increase was observed in rats older than 26 weeks^19^. Although this demonstrates an interaction between CVD and amyloid/tau, it remains unclear whether there is a direct relationship between amyloid/tau and CVD. From a management perspective, insights into the mechanism by which CVD contributes to the pathogenesis of AD will allow for the development of disease-modifying agents. To further clarify the impact of chronic CVD on amyloid and Tau pathways, we performed a graduated ligation of unilateral common carotid artery occlusion (UCCAO) in transgenic AD mice and quantified amyloid and Tau levels and additionally studied the role of autophagy in the syndrome of AD + CVD.

## Results

### UCCAO induces Tau phosphorylaion at Serine 199/202 in the hippocampus of young AD mice

To investigate the role of hypoxia in AD pathology, we first established an *in vivo* chronic cerebral hypoperfusion condition in both wild type (WT) and 3xTg-AD (AD) mice that carried the PS1_M146V_, APP_Swe_, and Tau_P301L_ mutants via applying unilateral common carotid artery ligation, which is also termed unilateral common carotid artery occlusion (UCCAO). The 3xTg-AD mice were generated by microinjecting two transgenes, APP_Swe_ and Tau_P301L_, into single-cell embryos from homozygous PS1_M146V_ knockin mice. Both the wild type (WT) and 3xTg-AD mice were originally generated as a hybrid 129/C57BL6 background^20^. These mice develop age-related, progressive neuropathology including plaques and tangles. Extracellular Aβ deposits are apparent by the sixth months in the frontal cortex, and become more extensive by twelfth months. Tau pathology is evident by twelfth months. Synaptic dysfunction and LTP deficits occur prior to plaques and tangles^20^,^21^.

**Figure 1:**
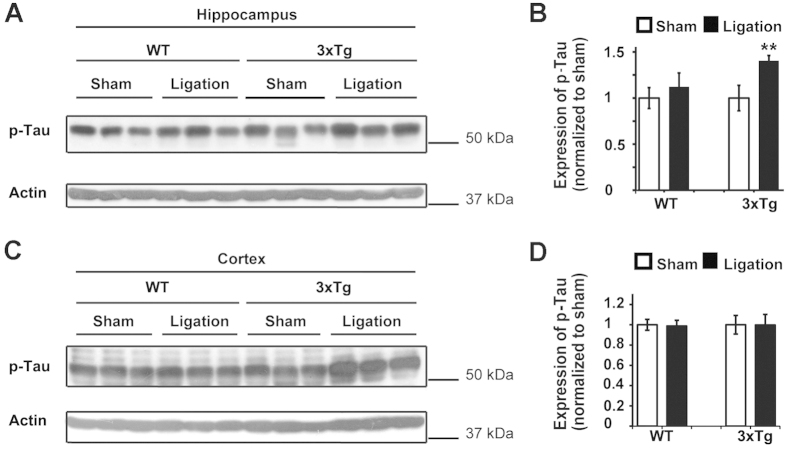
UCCAO ligation increases p-Tau expression in young 3xTg-AD mice. (**A**) UCCAO ligation was conducted on three-month-old WT and 3xTg-AD mice. Two months after ligation, protein lysates were extracted from the hippocampus of the ligated side and were subjected to western blot (WB) analysis with anti-phospho-Tau antibody. Actin was used as a loading control. Each line represents an individual mouse. (**B**) Quantification and statistical analyses of A. The mean ± SE of the relative protein level (normalized to sham) of phospho-Tau is shown. n = 3, Student’s *t*-test (two tailed), **p < 0.01. (**C**) UCCAO ligation was conducted on three-month-old WT and 3xTg-AD mice. Two months after ligation, protein lysates were extracted from cortex of the ligated side and were subjected to WB analysis with an anti-phospho-Tau antibody. Actin was used as a loading control. Each line represents an individual mouse. (**D**) Quantification and statistical analyses of C. The mean ± SE of relative protein level (normalized to sham) of phospho-Tau is shown. n = 5–6, Student’s *t*-test (two tailed). Cropped blots were presented in (**A**,**C**). The gels have been run under the same experimental conditions. Full-length blots were presented in Supplementary Figure 2.

Given that pathological phenotypes of amyloid beta (Aβ) deposition and the phosphorylation of Tau are hallmarks in AD^22^, we first investigated whether the level of phosphorylated Tau can be changed by chronic cerebral hypoperfusion to study the pathway by which cerebral hypoperfusion contributes to AD. Two month after ligation, brain tissue was harvested and lysed, and p-Tau was detected by anti-Tau phospho-Serine 199/202 antibody. We found that in young adult mice (3-month-old), two months after UCCAO ligation, the level of p-Tau was significantly increased in the hippocampus of AD mice compared with the sham AD (Fig. 1A,B, 40% increase); however, UCCAO ligation induced no change of p-Tau in the hippocampus of WT mice. Although we observed more p-Tau increased in the hippocampus of 3xTg-AD mice than the WT mice after UCCAO, there was no statistical significant difference of p-Tau in AD compared to WT animals post UCCAO (p = 0.068253). Moreover, both WT and AD mice showed no significant induction of p-Tau in the cortex of young mice, upon ligation (Fig. 1C,D).

In addition, we examined p-Tau expression from the contralateral hippocampus of the 3-month-old (young) UCCAO mice. We found that there is no obvious increase of p-Tau in the contralateral tissue of the UCCAO mice (Supplementary Figure S1A,B). Our observation correlates with the published studies on UCCAO model, that hypoperfusion is mainly happed on the ipsilateral side of the ligation^23^,^24^.

### UCCAO induces Tau phosphorylaion at Serine 199/202 in both aged WT mice and AD mice

**Figure 2:**
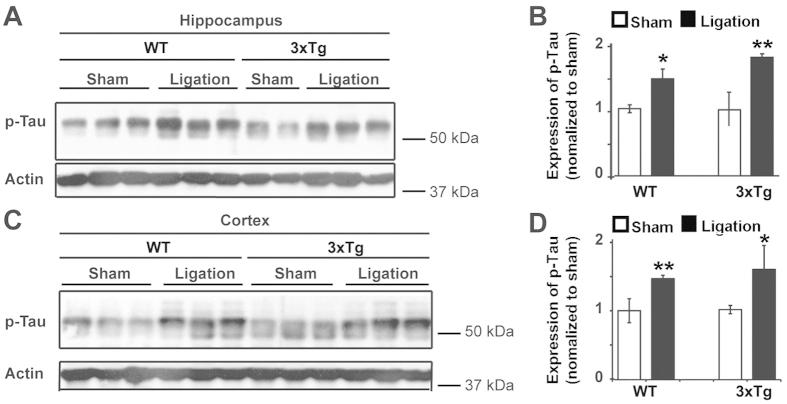
UCCAO ligation increases p-Tau expression in both aged WT and 3xTg-AD mice. (**A**) UCCAO ligation was conducted on sixteen-month-old WT and 3xTg-AD mice. Two months after ligation, protein lysates were extracted from the hippocampus of the ligated side and were subjected to WB analysis with anti-phospho-Tau antibody. Actin was used as a loading control. Each line represents individual mouse. (**B**) Quantification and statistical analyses of A. The mean ± SE of the relative protein level (normalized to sham) of phospho-Tau is shown. n = 5–6, Student’s *t*-test (two tailed), *p < 0.05; **p < 0.01. (**C**) UCCAO ligation was conducted on sixteen-month old WT and 3xTg-AD mice. Two months after ligation, protein lysates were extracted from the cortex of the ligated side and were subjected to WB analysis with anti-phospho-Tau antibody. Actin was used as the loading control. Each line represents individual mouse. (**D**) Quantification and statistical analyses of C. The mean ± SE of relative protein levels (normalized to sham) of phospho-Tau is shown. n = 5–6, Student’s *t*-test (two tailed), *p < 0.05; **p < 0.01. Cropped blots were presented in (**A**,**C**). The gels have been run under the same experimental conditions. Full-length blots were presented in Supplementary Figure 2.

Given that aging is an important risk factor for AD pathology, we then investigated the effect of hypoperfusion on p-Tau in aging mice. UCCAO ligation was applied to 16-month-old WT and AD mice. Two months after ligation, we examined whether ligation resulted in healthy aged rodents developing AD pathology and whether the AD brain would demonstrate an exaggerated AD pathology. We found that p-Tau was dramatically increased in both WT and AD mice (Fig. 2). Specifically, we found that in the hippocampus of WT mice, hypoperfusion induced a 45% increase in p-Tau expression compared to sham (Fig. 2A,B). The ligation-induced increase of p-Tau was even higher in AD mice (77% increase compared to AD sham) (Fig. 2A,B); however, there was no significant difference in Tau phosphorylation between the ligated AD mice and the ligated WT mice (p = 0.19988). A similar induction of p-Tau increase was also observed in the cortex of both WT and AD mice post ligation. In WT mice, there was 47% increase of p-Tau in ligated mice compared to sham ones. In AD mice, there was a 60% increase of p-Tau in ligated mice compared to sham (Fig. 2C,D). Although p-Tau has increased more in the cortex of AD mice than the WT mice (60% vs. 47%), there was no statistical significant difference of p-Tau in AD mice compared to WT mice post UCCAO (p = 0.33800). Our results suggest that chronic cerebral hypoperfusion led to the healthy aging brain developing increased Tau phosphorylation, consistent with AD pathology. Furthermore, chronic cerebral hypoperfusion led the aging AD brain developing a more severe p-Tau phenotype in both the hippocampus and cortex. Together, our results indicate that cerebral hypoperfusion results in an increase of p-Tau in mice brain. Importantly, both the genetic risk factor and aging can further potentiate hypoperfusion-induced p-Tau elevation.

### UCCAO does not affect Aβ_42_ level in WT and AD mice

**Figure 3:**
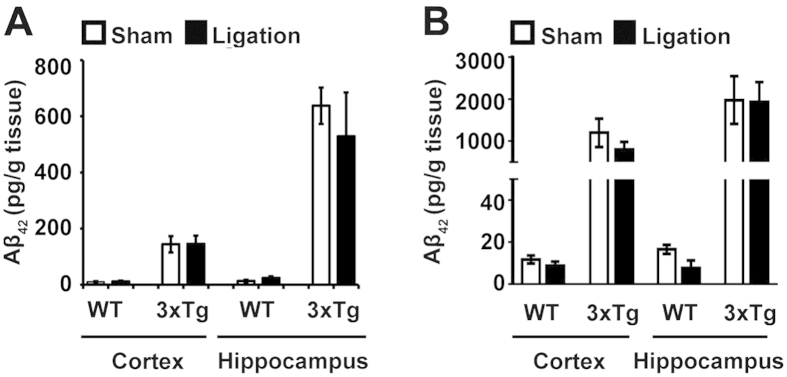
Aβ_42_was not elevated by UCCAO ligation in WT and 3xTg-AD mice. Expression of Triton-soluble Aβ_42_was detected using ELISA kits from Wako. (**A**) The expression of Aβ_42_in young mice (5 mth). (**B**) Expression of Aβ_42_in old mice (18 mth). The data are expressed as the mean ± SE (pg/g tissue); n = 3; Student’s *t*-test (two-tailed), not significant.

Because the accumulation of Aβ_42_ is another featured pathology in AD^22^, we next evaluated the expression of Aβ_42_ in the brain of ligated and sham mice by ELISA. We used Triton to lyse the brain tissue and to conduct the ELISA assay for Aβ measurement. This detergent-soluble Aβ detection method has shown to detect the increase of Aβ in AD brain^25^. Here, we found that, under sham conditions, the production of Aβ_42_ in both the hippocampus and cortex of AD mice was significantly higher than the WT mice (Fig. 3A; 144.11 pg/g tissue versus 10.31 pg/g tissue, in cortex; 637.57 pg/g tissue versus 13.73 pg/g tissue, in hippocampus); however, ligation had no effect on the expression of Aβ_42_ in either the hippocampus or cortex of both the WT and AD mice (Fig. 3A). Similar results were also obtained in 16-month-old mice (Fig. 3B). Although the production of Aβ_42_ was even higher in the aged AD mice than in the young AD mice, which is consistent with the previous observation of age-dependent Aβ accumulation in this mouse model^20^, ligation still had no significant effect on the generation of Aβ_42_ in the aged WT and AD mice, both in the cortex and hippocampus. To conclude, ligation had no significant effect on the expression of Aβ_42_ in either young or aged mice.

### UCCAO downregulates the autophagy pathway in the cortex of aged WT mice and AD mice

**Figure 4:**
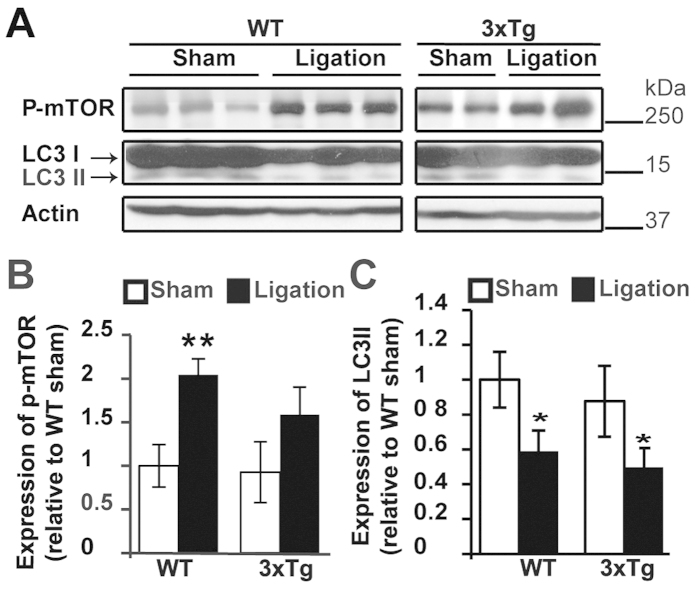
UCCAO ligation impaired autophagy activity. (**A**) UCCAO ligation was conducted on sixteen-month-old WT and 3xTg-AD mice. Two months after ligation, protein lysates were extracted from cortex of the ligated side and were subjected to WB analysis with anti-phospho-mTOR and anti-LC3 antibody. Actin was used as a loading control. Each line represents an individual mouse. (**B**) Quantification and statistical analyses of p-mTOR expression. The mean ± SE of relative protein levels (normalized to sham) of phospho-mTOR is shown. n = 3, Student’s *t*-test (two tailed), **p < 0.01. (**C**) Quantification and statistical analyses of LC3-II expression. The mean ± SE of the relative protein level (normalized to sham) of LC3-II is shown. n = 3, Student’s *t*-test (two tailed), *p < 0.05. Cropped blots were presented in (**A**). The gels have been run under the same experimental conditions. Full-length blots were presented in Supplementary Figure 2.

The autophagy pathway has been increasingly recognized as a key pathological mechanism in AD^26^,^27^,^28^. To further elucidate whether autophagy pathways are involved in cerebral hypoperfusion-induced AD development, we evaluated the autophagy activity change in response to hypoperfusion in both WT and AD mice. mTOR is an inhibitory signal in the autophagy pathway^29^. In particular, the increase of phospho-mTOR S2448 can inhibit the initiation of autophagosomes^30^. We found that phospho-mTOR S2448 is significantly increased in the cortex of aged WT mice two months after UCCAO ligation compared with sham mice (Fig. 4A,B). Consistently, LC3-II, which is a direct indication of autophagy activity^29^, was found to decrease significantly in the cortex of the ligated aged WT mice compared with the sham mice (Fig. 4A,C). The increased expression of phospho-mTOR S2448 and the decreased expression of LC3-II were also found in the ligated AD mice compared with the sham mice (Fig. 4). These results indicated that chronic UCCAO resulted in decreased autophagy activity in the cortex regardless of WT and AD mice.

### Autophagy activity is reduced in the frontal cortex of AD post-mortem tissues

**Figure 5:**
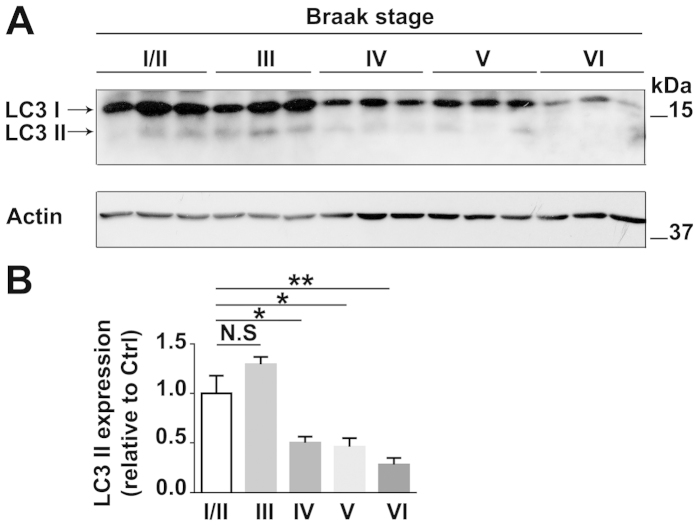
Autophagy activity is impaired in AD brain. (**A**) Protein lysates were extracted from the frontal cortex of human AD brain of various Braak stages (Braak stages III, IV, V, VI) and their age-matched healthy controls (Braak stage I/II). Protein lysates were subjected to WB analysis with anti-LC3 antibody. Actin was used as a loading control. Each line represents individual patient sample. (**B**) Quantification and statistical analyses of LC3-II expression in AD brain of various Braak stages and age-matched healthy controls. Data are presented as the mean ± SE of the relative protein level (normalized to controls) of LC3-II. n = 3 in each Braak stage, one-way ANOVA, Bonferroni test, *p < 0.05; **p < 0.01. Cropped blots were presented in (**A**). The gels have been run under the same experimental conditions. Full-length blots were presented in Supplementary Figure 2.

To further validate that autophagy activity deficits are involved in human AD pathology, we examined LC3 expression in the human AD frontal cortex. We found that the expression of LC3-II showed a significant decrease in expression in the frontal cortex of the late stage AD patients compared to the aged matched healthy controls (Braak stage I/II) (Fig. 5A,B). This suggests a defective autophagy activity in AD. The demographic information for AD and control post-mortem tissues is shown in Table 1.

## Discussion

In this study, we demonstrated that chronic cerebral hypoperfusion from graduated unilateral common carotid occlusion results in an increase in p-Tau levels in the hippocampus and cortex of both aged WT and AD mice; however, ligation had no effect on the expression of Aβ_42_ in either the hippocampus or cortex of the WT and AD mice. We additionally demonstrated that chronic cerebral hypoperfusion results in a decrease in autophagy activity in the cortex of both AD and WT mice.

Chronic cerebral hypoperfusion has been associated with cognitive impairment in AD. A moderate and persistent decrease in cerebral blood flow could compromise memory processes and deteriorate dementia^18^,^31^,^32^. The degree or location of hypoperfusion in mild cognitive decline has even been suggested to be a predictive marker for AD progression^33^,^34^. A unilateral occlusion model of UCCAO is one of the approaches to produce chronic cerebral hypoperfusion in mice and is thus ideal for investigating the contribution of chronic cerebral hypoperfusion to cognitive deficits and AD pathology^23^,^35^. Bilateral common carotid artery occlusion in rats has been well established as a model for studying chronic cerebral hypoperfusion-related neurodegenerative diseases^36^; however, this two vessel occlusion model causes severe ischemia damage in gerbils that lack a complete circle of Willis and in many mouse strains. A high mortality rate was also observed in our AD mice strains. Therefore, we performed a unilateral occlusion model to study hypoperfusion-related cerebral damage in AD mice.

Kitaguchi’s work used APP_SweInd_ mouse model of AD to study the bilateral carotid artery stenosis’s (BCAS) effect on AD pathology, and they only focused on the Aβ analysis and found chronic cerebral hypoperfusion increased Aβ deposition in APP_SweInd_ transgenic mice^37^; whereas, in Zhao’s created UCCAO in WT mice study, they found that an increased Tau phosphorylation in the brain after chronic cerebral hypoperfusion^23^. However, none of the above studies have investigated the impact of UCCAO in 3xTg-AD mice. Here, we provided novel findings to demonstrate that elevated p-Tau and reduced autophagy are major cellular changes associated with hypoperfusion in AD mice, whereas Aβ_42_ levels are not influenced by hypoperfusion.

In addition, we found enhanced p-Tau in the hippocampus compared with the cortex, which suggests that the hippocampus is more vulnerable to chronic cerebral hypoperfusion-induced Tau hyper-phosphorylation in AD. This correlates with the induction of hypoxia marker, hypoxia-inducible factor-1α (HIF-1α)’s expression and histological atrophy, that hippocampus is more susceptible than the cortex to hypoperfusion in the UCCAO model^23^,^38^. In addition, this differential vulnerability of Tau hyperphosphorylation is fully consistent with the fact that the hippocampus is more vulnerable to neurodegeneration than is the cerebral cortex in AD, further supporting the role of chronic cerebral hypoperfusion in AD. In addition, our study focused on young and aging AD mice and has shown that chronic hypoperfusion may accelerate AD neuropathology in a latent manner over an extended period of time via the enhancement of Tau phosphorylation^23^,^39^,^40^,^41^. This effect is likely to be more pronounced in older animals; however, it remains to be addressed in the future whether chronic cerebral hypoperfusion induces the abnormal phosphorylation of Tau protein would consequently accelerates synaptic dysfunction and cognitive deficits.

Several studies have reported that chronic ischemia/hypoxia mechanistically contributes to AD pathogenesis via the alteration of Aβ metabolism^37^,^42^. Surprisingly, we found no obvious changes in WT or AD mice upon chronic cerebral hypoperfusion compared to the corresponding sham-operated group. The underlying mechanisms are not clear and need to be further elucidated. Although we did not demonstrate the chronic cerebral hypoperfusion resulted in the enhancement of Aβ accumulation in WT and AD mice, our finding is correlated with the clinical study of the interrelationships between vascular disease pathology and amyloid pathology, which suggests that amyloid and vascular pathologies seem to be at least partly independent processes that affect cognitive decline in the elderly^12^. The observation of no Aβ change in AD mice in response to hypoperfusion is correlated with the clinical amyloid PET that the impact of CVD in AD is independent of amyloid. This suggests that CVD may contribute, via non-amyloid pathways, in the pathogenesis of dementia.

Autophagy is an important degradation pathway for the removal of damaged organelles and macromolecules^43^. The initiation of this process requires the inhibition of mTOR activity^29^. mTOR is a key protein kinase that regulates cellular metabolism in response to nutrient and growth hormones^30^,^44^. Animal experiments in various species have identified mTOR as a central regulator of aging^28^,^30^,^45^. Consistently, autophagy activity declines with age and is deficient in the early stage of AD^27^,^45^,^46^,^47^. As aging is the most important risk factors of AD, it is highly speculative that mTOR and autophagy play a central role in AD etiology. Here, we showed chronic cerebral hypoferfusion can induce a strong mTOR activity upregulation and autophagy deficiency in both WT and AD mice (Fig. 4). Because more than 60% of the AD patients suffer from cerebral hypoperfusion^13^,^18^, our findings implicate that autophagy deficiency is highly likely to be a primary cause of the progression of AD. It is possible that chronic cerebral hypoperfusion, which alters the metabolism status of the brain, results in autophagy deficiency and in turn potentiates the onset of AD. Nevertheless, the interrelationship among autophagy, Aβ and p-Tau is still not clear. The accumulation of Aβ can increase mTOR signaling activity and in turn impairs autophagy activity^46^; however, we have here shown that the autophagy pathway was impaired by chronic hypoperfusion without Aβ accumulation. This indicates that autophagy impairment may be an early and primary deficit, which is independent of Aβ pathology, in chronic hypoperfusion-induced AD. It has also been reported that rapamycin, mTOR’s inhibitor, can ameliorate Tau pathology by increasing autophagy activity in 3xTg-AD mice^46^. Whether mTOR inhibition or autophagy activation can inhibit chronic hypoperfusion-induced p-Tau elevation will be a focus of future study.

Compared to the enhancement of p-Tau is more susceptible in the hippocampus than the cortex after UCCAO (Figs 1 and 2), we observed that autophagy deficiency is more susceptible in the cortex than the hippocampus post UCCAO (Fig. 4). This is plausible because a significantly reduced cerebral blood flow was also observed in the ipsilateral cerebral cortex in the UCCAO model^38^. In addition, in mice, we noticed that there is no significant difference of LC3-II expression between the 3XTg-AD mice and that in their age-matched WT mice before the UCCAO (Fig. 4). By contrast, in human autopsy samples, the expression of LC3-II is significantly lower in AD brain than that in the age-matched controls (Fig. 5). This may indicate that AD mice carrying APP, PS1, and Tau genetic mutations are not sufficient to cause autophagy deficit within a short life span in mouse. In fact, in an animal model, pathological changes are studied over a relatively short period of time; whereas in human, AD starts from lesions occurring and evolving over the course of many years or decades.

The finding that elevated p-Tau is a direct consequence to chronic cerebral hypoperfusion is of high clinical relevance. Although Tau changes have been demonstrated in human studies following ischemic injury, it was not possible to ascertain whether the Tau increase was a direct consequence of ischemia and hypoperfusion. In this animal study, we were able to demonstrate that ischemia and hypoperfusion directly results in an increase in p-Tau. Longitudinal human studies have demonstrated that CVD may be an early process in the pathogenesis of AD, and our findings which showed an increase in p-Tau occured in both young and aged AD mice further supports this observation^48^. With the recent availability of anti-Tau agents and human clinical trials with anti-Tau agents, our finding may be useful in planning animal and human clinical trials for the syndrome of AD + CVD^49^.

## Materials and Methods

### Mice

The 3xTg-AD mice were purchased from Jackson’s laboratory, which were generated by microinjecting two transgenes, APP_Swe_ and Tau_P301L_, into single-cell embryos from homozygous PS1_M146V_ knockin mice. Both the wild type (WT) and 3xTg-AD mice were originally generated as a hybrid 129/C57BL6 background^20^. These mice develop age-related, progressive neuropathology including plaques and tangles^20^,^21^. All uses of experimental animals were conducted with protocols that were approved by the Institutional Animal Care and Use Committee (IACUC) of the National Neuroscience Institute. Methods involving mice were carried out in accordance with the approved guidelines. Male 3xTg-AD mice and their age/gender matched control WT mice (3-month-old and 16-month-old) were used.

### Post-mortem brain tissues

We obtained sporadic AD and control post-mortem samples from Bryan Alzheimer’s Disease Research Center (Bryan ADRC), Duke University. Post-mortem study was approved by the Singhealth IRB Committee. The methods were carried out in accordance with the approved guidelines and regulations.

### Cerebral hypoperfusion model generation

Unilateral common carotid artery occlusion (UCCAO) surgery was conducted in 3xTg-AD and WT mice to generate hypoxia. Mice of 3 months and 16 months of age were used for the surgery. Briefly, mice were anesthetized with ketamine (60 mg/kg) and xylazine (5 mg/kg) intraperitoneally. Midline neck incisions were made and the left common carotid artery (CCA) was isolated by removing surrounding connective tissue and nerves. The CCA was ligated with non-absorbable 6/0 black braided silk suture (Cat# SP102, LOOK). Sham-operated mice underwent similar procedures without the ligation of the CCA. Two months after the UCCAO surgery, brain tissues of ligated mice and sham control mice were harvested for AD-related pathology detection.

### Western blot

Total protein was extracted from the left (ipsilateral) cerebral cortex and hippocampus by using RIPA buffer that contained phosphatase and protease inhibitors. The lysates were separated by electrophoresis on an SDS-PAGE gel and transferred onto PVDF membrane (Millipore). The blot was probed overnight with primary antibodies, including rabbit anti-phospho-Tau (Ser199/202) (1:1000, Millipore), mouse anti-LC3 (1:200, Nano tools), rabbit anti-BDNF (1:3000), rabbit anti-phospho-mTOR (Ser2448) (1:1000, Cell signaling), and mouse anti-β-actin (1:2000, Santa Cruz) at 4°C. HRP-labeled secondary antibodies (1:3,000, GE life Science) were used and the signals were visualized using an ECL kit (GE Healthcare).

### ELISA

Aβ_42_ was detected using an ELISA kit from Wako (Cat# 292–64401). Briefly, brain tissue was lysed using lyses buffer containing 1% Triton, phosphatase and protease inhibitors. Brain tissue was homogenized by passing through 26 gauge needles. After centrifuged at 13000 rpm for 15 minutes, the supernatant which contained the water-soluble and Triton-soluble Aβ_42_ were dispensed onto ELISA plate wells. The plates were sealed and kept at 4 °C overnight. On the next day, we discarded the solution inside the wells and washed the wells with wash solution for five times. Then, we dispensed 100 μl HRP-conjugated antibody solutions into the wells, sealed the wells and let the wells stand at 4 °C overnight. On the third day, we removed the antibody solution from the wells and washed the wells with wash solution five times. Finally, we added 100 μl TMB solutions to the wells and started the reaction at room temperature in the dark for 30 min before adding the stop solution. We then read the absorbance of each well at 450 nm using a Tecan microplate reader.

### Statistical analysis

In each experimental condition, 3–6 mice were used. Quantitative data were expressed as the mean ± SE. Statistical analyses were conducted using Student’s t-test for two group comparison and by one-way ANOVA for multiple group comparison. Post hoc Bonferonni test was used after a one-way ANOVA analysis. Two-way ANOVA was used to test whether age and treatment are significant factors. Statistical significance was defined when ***P < 0.001, **P < 0.01, *P < 0.05.

## Additional Information

**How to cite this article**: Qiu, L. *et al.* Chronic cerebral hypoperfusion enhances Tau hyperphosphorylation and reduces autophagy in Alzheimer's disease mice. *Sci. Rep.* **6**, 23964; doi: 10.1038/srep23964 (2016).

## Supplementary Material

Supplementary Information

## Figures and Tables

**Table 1:** Demographic information for AD post-mortem tissues.

Patient ID	Gender	Tissue type	Diagnoses (B & B stage)	Diagnoses (AD)	Age	PMD (Hrs)	Genetic Risk (ApoE)
913	F	Frontal Cortex	I	Control	67	8	33
787	F	Frontal Cortex	I	Control	90	10.63	33
591	F	Frontal Cortex	I	Control	78	5.52	33
568	M	Frontal Cortex	III	AD	88	1.25	34
645	F	Frontal Cortex	III	AD	80	5.75	34
760	F	Frontal Cortex	III	AD	77	4	44
1222	F	Frontal Cortex	IV	AD	82	2.75	34
1342	F	Frontal Cortex	IV	AD	88	3.5	33
1594	M	Frontal Cortex	IV	AD	88	3.92	33
910	F	Frontal Cortex	V	AD	79	4.2	44
1111	M	Frontal Cortex	V	AD	78	8	34
1259	F	Frontal Cortex	V	AD	79	8	44
1526	F	Frontal Cortex	VI	AD	88	2	34
1254	F	Frontal Cortex	VI	AD	82	10.25	34
1679	M	Frontal Cortex	VI	AD	67	7	33

AD: Alzheimers disease; B & B stage: Braak stage; PMD: postmortem delay.
