# Supplementary material for: Chronic cerebral hypoperfusion enhances Tau hyperphosphorylation and reduces autophagy in Alzheimer’s disease mice
Source: Sci Rep. 2016 Apr 6;6:23964. doi: 10.1038/srep23964 (PMC4822118; doi:10.1038/srep23964)
Supplement: Supplementary Information [file srep23964-s1.doc]

**Chronic cerebral hypoperfusion enhances Tau hyperphosphorylation and reduces autophagy in Alzheimer’s disease mice**

Lifeng Qiu^1^, Gandi Ng^2^, Eng King Tan^3,4,5^, Ping Liao^2*^, Nagaendran Kandiah^6*^, Li Zeng^1,5*^

^1^Neural Stem Cell Research Lab, Research Department, National Neuroscience Institute, 11 Jalan Tan Tock Seng, Singapore 308433.

^2^Calcium Signaling Laboratory, Research Department, National Neuroscience Institute, 11 Jalan Tan Tock Seng, Singapore 308433.

^3^Department of Neurology, National Neuroscience Institute, SGH Campus, Singapore 169856.

^4^Research Department, National Neuroscience Institute, 11 Jalan Tan Tock Seng, Singapore 308433.

^5^Neuroscience & Behavioral Disorders Program, DUKE-NUS Graduate Medical School, Singapore 169857.

^6^Neurology Department, National Neuroscience Institute, 11 Jalan Tan Tock Seng, Singapore 308433.

*** Corresponding Authors:**

Ping Liao, [ping_liao@nni.com.sg](mailto:ping_liao@nni.com.sg)

Nagaendran Kandiah, nagaendran_kandiah@nni.com.sg

Li Zeng, [Li_Zeng@nni.com.sg](mailto:Li_Zeng@nni.com.sg)

**Running Title:** Hypoxia phosphorylates Tau in AD development

**Keywords:** Alzheimer’s disease; Tau; Amyloid beta; Phosphorylation; Unilateral common carotid artery occlusion (UCCAO) ligation; Cerebral hypoperfusion; Hypoxia; Autophagy

**Supplementary information:**

**Supplementary Figure 1**


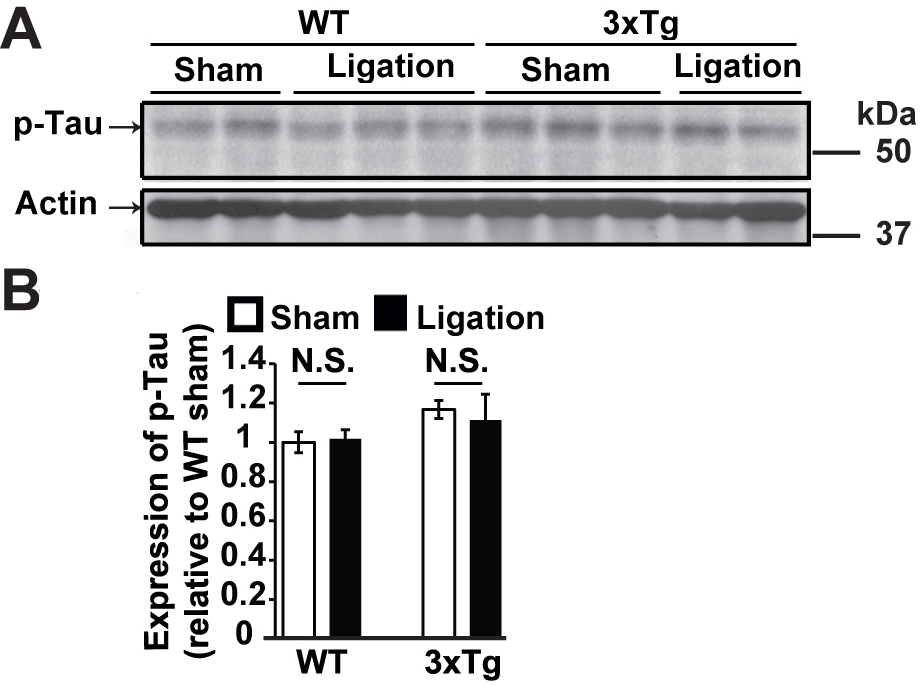


**Supplementary Figure 1.** **UCCAO caused no change of p-Tau expression in the contralateral hippocampus**. (**A**) Western blot showed expression of p-Tau in the contralateral side hippocampus of the 3-month-old WT and 3xTg-AD mice. Actin was used as a loading control. Each line represents an individual mouse sample. Compared with age-matched sham animals, UCCAO ligation didn’t change the expression of p-Tau in the contralateral side hippocampus of the ligated WT and 3xTg-AD mice. (**B**) Quantification and statistical analyses of p-Tau expression in the contralateral hippocampus in response to UCCAO. Data was presented as mean ± SE of relative protein levels (normalized to sham). n=3, Student’s *t*-test (two tailed) showed there is no significant difference between the ligated and sham-operated mice. Cropped blots were presented in (**A**). The gels have been run under the same experimental conditions. Full-length blots were presented in **Supplementary Figure 2.**

**Supplementary Figure 2**


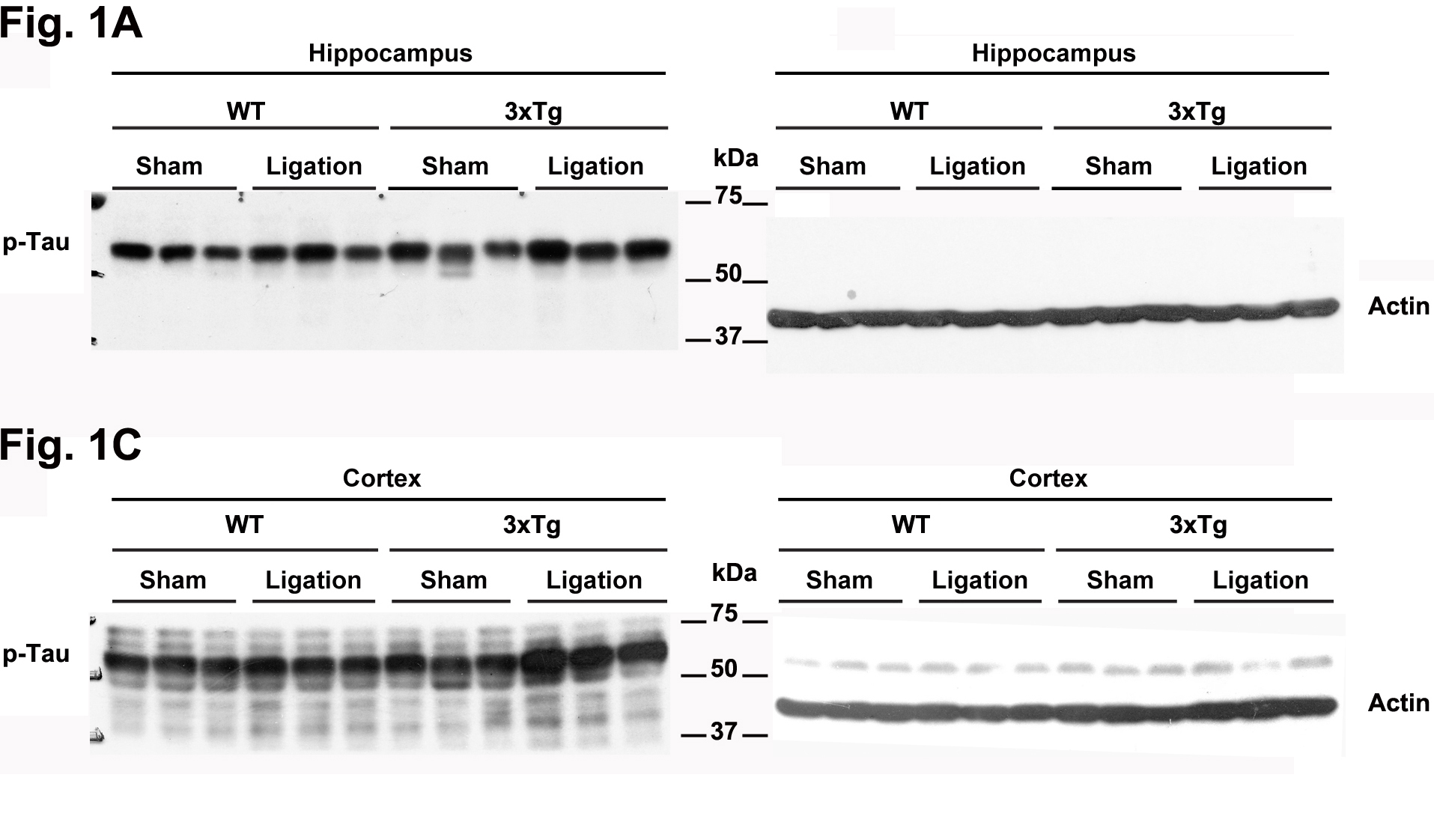


**
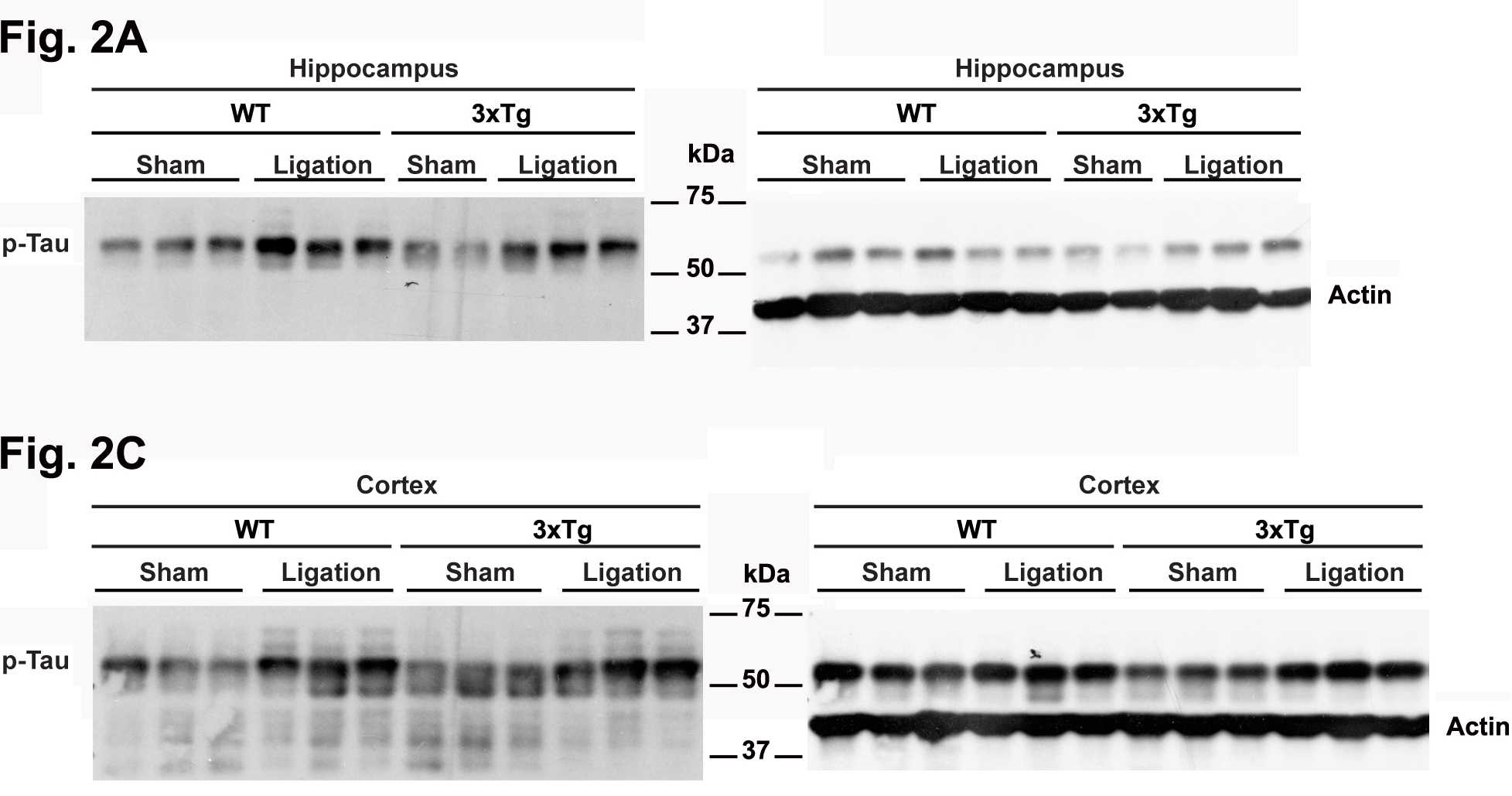
**


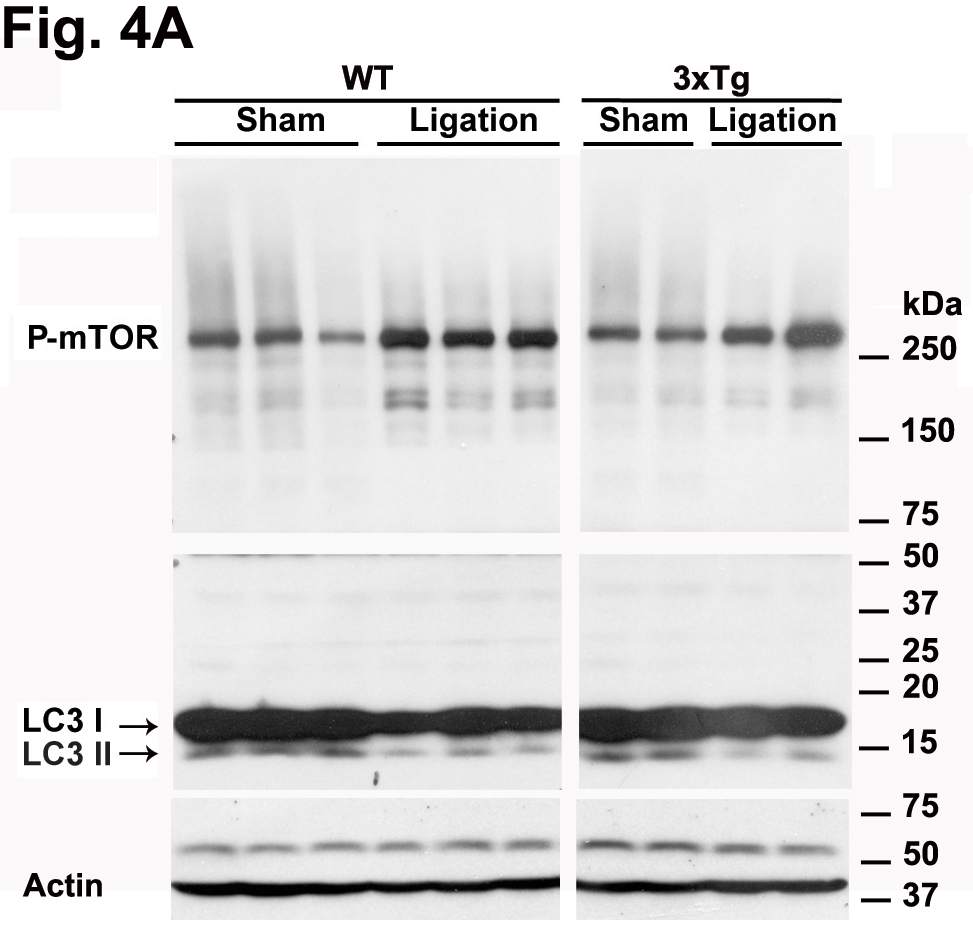


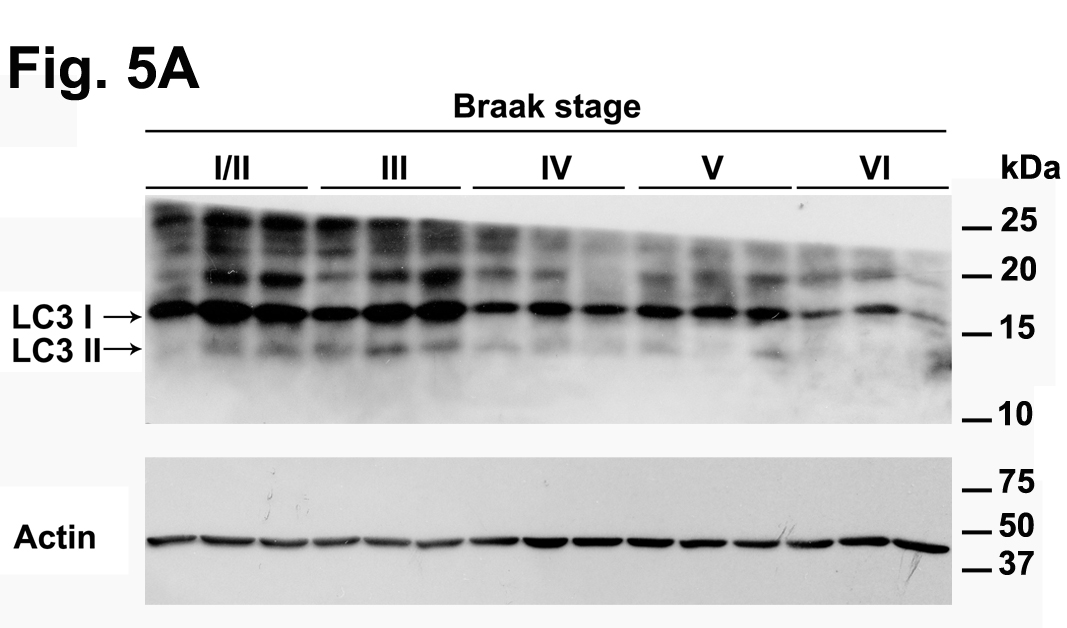


**
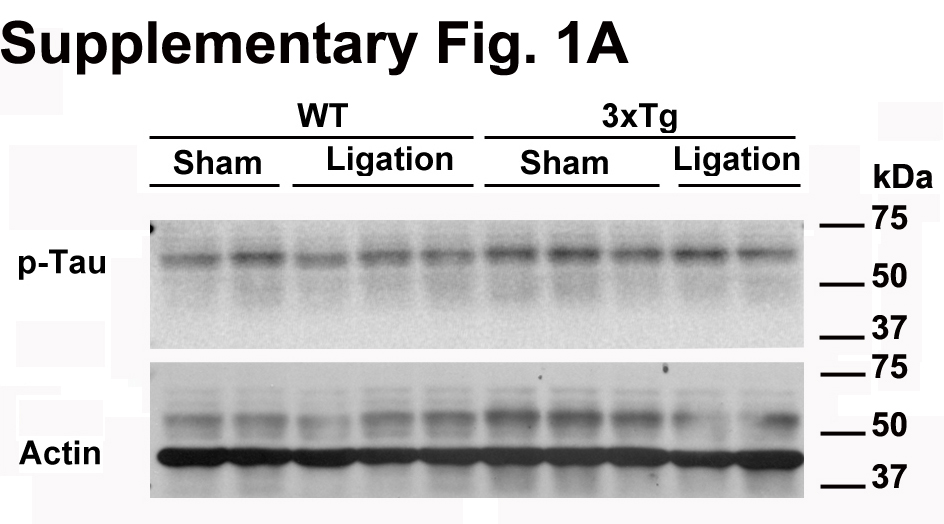
**

**Supplementary Figure 2**. **Full scans of the immunoblots presented in the paper.** The rectangles indicate the images presented in the paper.
